# Differential associations of diet with hepatic and muscle insulin resistance: insights from an dietary pattern analysis in the PERSON study

**DOI:** 10.1007/s00394-026-03996-8

**Published:** 2026-05-26

**Authors:** Axelle Hoge, Anne-Françoise Donneau, Nadia Dardenne, Michèle Guillaume, Lydia A. Afman, Edith J. M. Feskens, Gijs H. Goossens, Ellen E. Blaak

**Affiliations:** 1 Departement of Public Health, Liège University, Liège, Belgium; 2 Division of Human Nutrition and Health, Wageningen University, Wageningen, The Netherlands; 3 Department of Human Biology, Institute of Nutrition and Translational Research in Metabolism (NUTRIM), Maastricht University Medical Center+, Maastricht, The Netherlands

**Keywords:** Dietary pattern, Insulin resistance, Diabetes, Reduced rank regression

## Abstract

**Purpose:**

The relationship between dietary patterns (DPs) and type 2 diabetes is well established, but the potential role of tissue-specific insulin resistance (IR) in this association remains unclear. This study aimed to derive DPs using reduced rank regression (RRR), incorporating hepatic IR index (HIRI) and muscle insulin sensitivity index (MISI) as response variables. We also examined whether these patterns are associated with insulin sensitivity and pancreatic β-cell function.

**Methods:**

We conducted a cross-sectional analysis of 700 adults with overweight or obesity participating in the screening phase of the PERSON study. Dietary intakes were assessed using a food frequency questionnaire. RRR was used to derive DPs based on HIRI and MISI. Associations with HOMA-IR, HOMA-β, Matsuda index and Disposition index were tested using multiple regression models adjusted for socio-demographic and lifestyle factors.

**Results:**

One DP was retained, explaining 13.7% of the variation in HIRI, 2.8% in MISI, and 8.2% of the combined variation. This DP was characterized by high intakes of unprocessed red meat, processed meat, fresh cream and whipped cream, and low intakes of fruits, vegetables, and tea. It was significantly associated with higher HOMA-IR (β-coefficient ± SE: 0.04 ± 0.02) and HOMA-β (0.05 ± 0.01), and lower Matsuda index (− 0.08 ± 0.02).

**Conclusion:**

The identified DP was more strongly associated with hepatic than muscle IR. This finding highlights differential associations between diet and tissue-specific IR, and supports the relevance of considering tissue-specific insulin resistance phenotypes when investigating the relationship between diet, insulin resistance and type 2 diabetes risk.

*Trial registration* The trial was registered at https://clinicaltrials.gov/study/NCT03708419 (identifier NCT03708419).

**Supplementary Information:**

The online version contains supplementary material available at 10.1007/s00394-026-03996-8.

## Introduction

Whole-body insulin resistance (IR) is a pathological condition involving an impaired biological response to insulin in target tissues, and represents a major risk factor for type 2 diabetes and other cardiometabolic diseases [1–3].

Among modifiable determinants, diet plays a key role in glucose homeostasis through mechanisms that extend beyond weight loss [4–6]. There is convincing evidence for the role of dietary macronutrient composition and specific food groups in the development of IR [5–8]. However, humans generally do not consume single nutrient or food group, and the importance of diet in health may be better described by overall dietary patterns (DPs) [9–11].

DPs have drawn significant attention in the past decade because they reflect how foods are consumed in combination and capture the cumulative contribution of multiple foods and nutrients, as well as their potential interactions and intercorrelations within the overall diet [12–14]. Reduced rank regression (RRR) is a mixture of a hypothesis-oriented and a posteriori data-driven approach, aiming to identify food group combinations that explain a maximum of variation in pre-defined response variables [15]. These response variables are typically considered hypothetical intermediate outcomes of the disease of interest, allowing the evaluation of hypotheses on the pathways linking diet to disease through these response variables [15, 16]. The RRR method is particularly suitable for exploring how diet relates to type 2 diabetes through specific metabolic pathways.

Previous studies have investigated the effects of RRR-derived dietary patterns on type 2 diabetes and other IR-related diseases by using biomarkers such as fasting glucose and HOMA-IR [17]. However, it remains unknown whether DPs are related to tissue-specific measures of IR.

The liver and skeletal muscle are key organs in the regulation of whole-body glucose homeostasis. Under normal conditions, insulin suppresses hepatic glucose production while stimulating glucose uptake and storage in skeletal muscle, thereby maintaining healthy blood glucose levels across fasting and postprandial states.

In obesity, these coordinated metabolic processes become impaired. The liver shows a reduced ability to suppress glucose output, particularly in the fasting and early postprandial phase, while skeletal muscle exhibits a diminished capacity for insulin-stimulated glucose disposal. In parallel, disturbances in lipid handling and ectopic lipid accumulation in both tissues further contribute to impaired insulin signaling [18–20]. Importantly, insulin resistance does not necessarily develop uniformly across tissues. Individuals with overweight or obesity may present predominantly hepatic or muscle IR, reflecting distinct metabolic phenotypes that may differ in their cardiometabolic profiles and in their sensitivity to dietary exposures [21, 22].

Tissue-specific IR—liver IR (LIR) and muscle insulin resistance (MIR)—can be estimated from oral glucose tolerance test, using two indices that have been validated against the gold standard hyperinsulinemic-euglycemic clamp technique, namely the hepatic IR index (HIRI) and muscle insulin sensitivity index (MISI) [22–24].

The objective of the present study was to derive dietary patterns that are linked to HIRI and MISI (response variables) using a RRR approach in adults with overweight or obesity participating in the PERSonalized glucose Optimization through Nutritional intervention (PERSON) study. Secondly, we investigated whether the dietary patterns identified are associated with surrogate measures of insulin sensitivity and pancreatic β-cell function (HOMA-IR, Matsuda index, HOMA-β and disposition index). Our hypothesis is that diet may related to type 2 diabetes through metabolic pathways involving tissue-specific insulin resistance, with potential differences between hepatic and muscle phenotypes depending on dietary composition.

## Methods

### Study design

This is a cross-sectional analysis of the data collected at the screening visit of the PERSonalized glucose Optimization through Nutritional intervention (PERSON) study, a two-center 12-week dietary intervention study with a randomized, double-blind, controlled, parallel design. This study was designed to investigate the effects of an optimal versus sub-optimal dietary macronutrient intervention according to tissue-specific IR phenotype on glucose metabolism and other health outcomes. Further details of the rationale, recruitment procedures and data collection methods have been described elsewhere [21].

The PERSON study was carried out from May 2018 until November 2021 at Maastricht University Medical Center+ (MUMC+) and Wageningen University & Research (WUR), the Netherlands. The protocol was approved by the Medical Ethics Committee of MUMC+ (NL63768.068.17), registered at ClinicalTrials.gov (identifier NCT03708419), and conducted according to the principles of the Declaration of Helsinki. All subjects provided written informed consent before the start of the study.

### Study population

Subjects (age 40–75 years, body mass index (BMI) 25–40 kg/m^2^) were recruited in the vicinity of Maastricht and Wageningen on a volunteer basis through advertisements in local media and social networks. If potential participants met initial inclusion criteria based on a telephone pre-screening (e.g. age, weight, general health status and medication use), they were invited for a screening visit to fully assess eligibility for the PERSON trial, as previously described [21].

The present study was conducted using data from the screening phase of the PERSON study and therefore includes a broader population than the participants enrolled in the randomized controlled trial. While the RCT only included individuals presenting a predominant MIR or LIR phenotype, the screening dataset also includes individuals without a distinct MIR or LIR phenotype, as well as those presenting a mixed phenotype, who were not eligible for participation in the intervention study.

During screening, several exclusion criteria were applied, including pre-diagnosed type 2 diabetes, diseases or medication affecting glucose or lipid metabolism, major gastrointestinal diseases, history of major abdominal surgery, uncontrolled hypertension, smoking, alcohol consumption > 14 units/week, and > 4 h/week of moderate-to-vigorous physical activity.

**Fig. 1:**
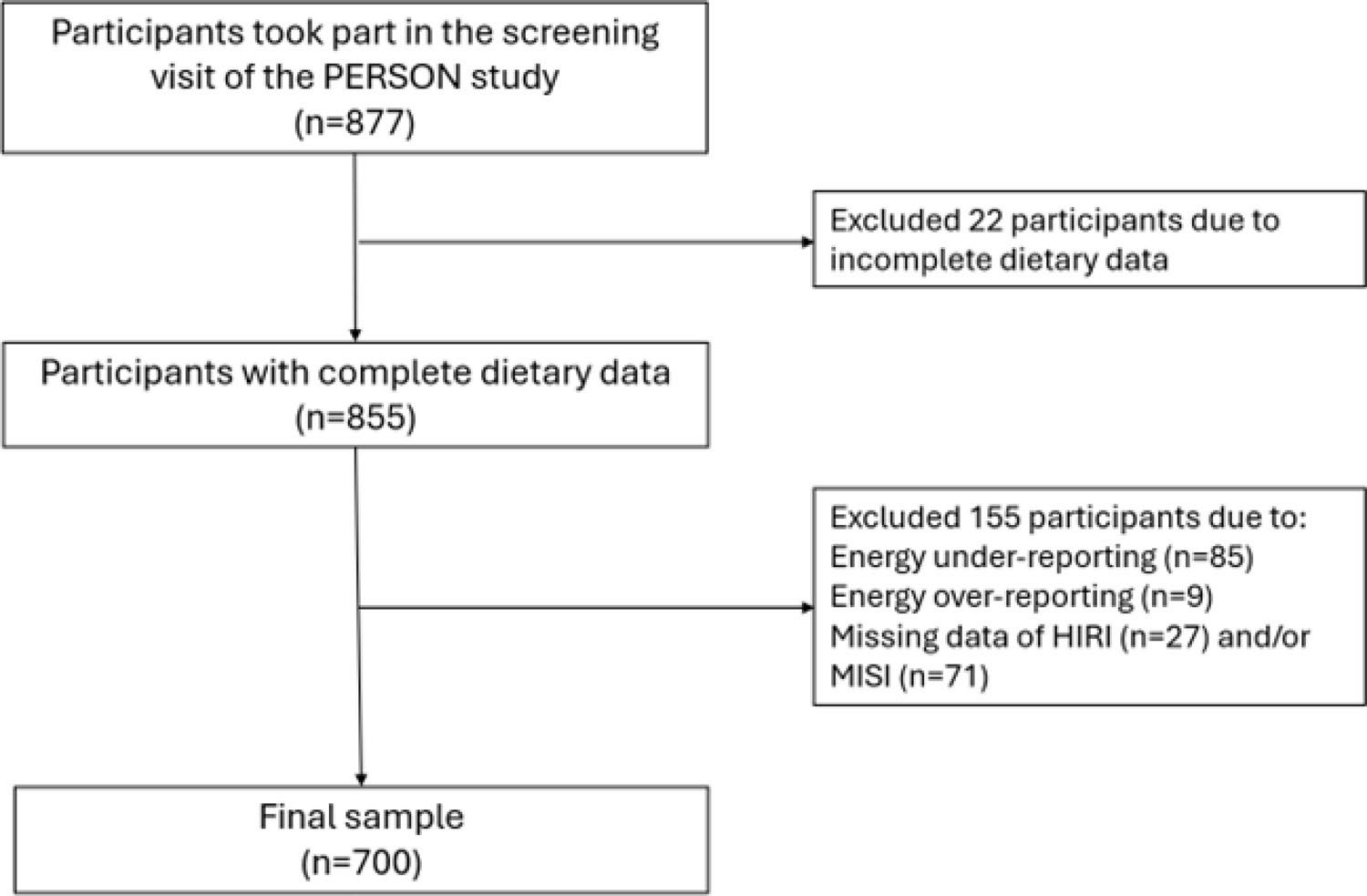
Flowchart of the sample selection. HIRI: the hepatic insulin resistance index; MISI: muscle insulin sensitivity index

As presented in Fig. 1, from the 877 participants fully screened, only those with complete dietary data were enrolled (*n* = 855) in the current study. Participants with under-reporting (*n* = 85) or over-reporting (*n* = 9) dietary intakes, and those with missing data of HIRI (*n* = 27) and/or MISI (*n* = 71) were excluded from data analysis. Dietary misreporting was evaluated by Goldberg’s method, using the ratio of daily energy intake (EI) to estimated basal metabolic rate (BMR) [25, 26]. A EI/BMR ratio < 0.87 and > 2.75 were used to identify energy under- and over reporters, respectively. This resulted in a final sample of 700 subjects. No formal sample size calculation was performed for the present analysis, as the study population was determined based on the primary objectives of the PERSON study. Specifically, the sample size was defined to detect expected differences in disposition index improvement between participants receiving their hypothesized optimal diet and those receiving their hypothesized suboptimal diet [21].

### Data collection and measurements

#### Sociodemographic and lifestyle characteristics

Data on sociodemographic aspects, medical history, medication use, and lifestyle were collected by a general questionnaire. Education level was categorized into low (no education, primary education, lower or preparatory vocational education, lower general secondary education), medium (intermediate vocational education, higher general senior secondary education or pre-university secondary education) and high (higher vocational education, university).

Study participants were asked to refrain from alcohol and vigorous physical activity 24 h prior to the visit and arrive in the morning after a > 10 h overnight fast. Body weight and height were measured twice without shoes or heavy clothing, recorded to the nearest 0.1 kg and 0.1 cm, respectively. Waist circumference was measured twice, using a non-flexible measuring tape, with precision to the nearest 0.1 cm. BMI was calculated using the standard formula of body weight in kilograms divided by the square of height in meters (kg/m^2^). Blood pressure readings were taken three times on the non-dominant arm using an automated sphygmomanometer after the subject has rested in a supine position for five minutes. The initial measurement served to acclimate the subject and was therefore excluded from the final analysis.

#### Dietary intake assessment and food group construction

Baseline habitual dietary intakes were estimated in all participants using a validated semiquantitative food questionnaire [27]. The participants reported their consumption of 163 food items over the previous month (4 weeks). For main food items, information of frequency (options ranged from “not in this month”, “1 day per month” to “7 days per week”) and quantity (in units or specified portion size) of consumption was asked. For secondary items (e.g. type of bread), the consumption frequency was reported using “never”, “sometimes”, “often”, and “always” statements. Responses were then converted to food intake in grams per day. Based on these data energy and nutrient intake were calculated using the 2006 Dutch Food Composition Table (NEVO) [28].

To perform dietary pattern (DP) analysis (also see *‘Statistical analysis’* below), food items were aggregated into 40 broader food groups according to (i) their nutritional similarities; (ii) the evidence base for relationships between food groups and health outcomes specifically with interest to blood glucose homeostasis [10, 11, 17, 29–32]; and (iii) the 2015 Dutch food-based dietary guidelines [33, 34] (see Supplementary Table 1).

#### Energy adjustment and handling of dietary misreporting

Food groups intakes were treated as adjusted for energy intake using the residuals method of Willet and Stampfer [35]. Given the well-documented risk of dietary misreporting in populations with overweight or obesity, we implemented several strategies to minimize this bias. First, we applied an energy misreporting correction by excluding individuals identified as under- or over-reporters of total energy intake (see study population). Additionally, all dietary variables included in the RRR models were energy-adjusted to account for variations in total energy intake. Finally, BMI was used as an adjustment factor in the 4th linear model related to the association between the DP score and measures of insulin sensitivity and pancreatic β-cell function. While these steps help reduce the impact of misreporting, some degree of measurement error remains possible.

#### 7-point oral glucose tolerance test

##### Tissue-specific IR indexes (HIRI and MISI)

Tissue-specific IR indexes were assessed by a 7-point oral glucose tolerance test (OGTT). Subjects were instructed to ingest 200 ml of a ready-to-use 75 g glucose solution (Novolab) within 5 min, and blood samples were collected from the antecubital vein via an intravenous cannula under fasting conditions (t = 0 min) and after ingestion of the glucose drink (t = 15, 30, 45, 60, 90, and 120 min) for determination of plasma glucose and insulin concentrations. At each time point, 2 ml of blood is discarded prior to drawing the blood samples. HIRI and MISI were estimated using the methods of Abdul-Ghani and colleagues [22]. The MISI calculation has been optimized using the cubic spline method [23]. HIRI and MISI were calculated according to the following formulas:

$$ {\mathrm{HIRI}} = {\mathrm{glucose}}\;0{-}{\mathrm{3}}0\left[ {{\mathrm{AUC}}\;{\mathrm{in}}\;{\mathrm{mmol}} = {\mathrm{L}} \times {\mathrm{h}}} \right] \times {\mathrm{insulin}}\;0{-}{\mathrm{3}}0\left[ {{\mathrm{AUC}}\;{\mathrm{in}}\;{\mathrm{pmol}} = {\mathrm{L}} \times {\mathrm{h}}} \right] $$

$$ {\text{MISI = (dGlucose/dt)/insulin[mean}}\;{\mathrm{during}}\;{\mathrm{OGTT}}\;{\mathrm{in}}\;{\text{pmol = L]}} $$

here dGlucose/dt is the rate of decay of plasma glucose concentration during the OGTT, calculated as the slope of the least square fit to the decline in plasma glucose concentration from peak to nadir. The decline in plasma glucose concentration after 60 min primarily reflects glucose uptake by peripheral tissues, mainly skeletal muscle.

MISI calculation may be not possible or possibly not biologically meaningful due to either a peak at 120 min, a “flat” curve, or nonnegligible rebound [23]. Both indexes were developed and validated against gold standard hyperinsulinemic-euglycemic clamp studies [22, 23].

The highest tertile of HIRI corresponds to individuals with LIR while the lowest tertile of MISI corresponds to individuals with MIR.

##### Glucose metabolism

Glucose status was defined according to WHO criteria [36]: normal glucose tolerance (NGT), fasting glucose < 5.6 mmol/L and 120-min glucose < 7.8 mmol/L; impaired fasting glucose (IFG), fasting glucose 5.6–6.9 mmol/L and 120-min glucose < 7.8 mmol/L; impaired glucose tolerance (IGT), fasting glucose < 5.6 mmol/L and 120-min glucose 7.8–11.0 mmol/L; combined IFG/IGT, fasting glucose 5.6–6.9 mmol/L and 120-min glucose 7.8–11.0 mmol/L; type 2 diabetes mellitus (T2DM), fasting glucose ≥ 7.0 mmol/L and/or 120-min glucose ≥ 11.1 mmol/L.

##### Measures of whole-body insulin sensitivity and pancreatic β-cell function

Homeostasis model assessment of insulin resistance (HOMA-IR) and β-cell function (HOMA-β) were calculated as follows: HOMA-IR = (fasting glucose [mmol/L] × fasting insulin [mU/L])/22.5 [37], and HOMA-β = (20 × fasting insulin [mU/L])/(fasting glucose [mmol/L] – 3.5).

Matsuda index was defined as: [10,000 ÷ square root of [fasting plasma glucose (mmol/l) × fasting insulin (pmol/l)] × [mean glucose (mmol/l) × mean insulin (pmol/l)]], using glucose and insulin values of time points 0, 30, 60, 90, and 120 min [38]. Disposition index was calculated as: [Matsuda index ∗ (AUC30min insulin/AUC30min glucose)], where AUC30min is the area under the curve between baseline and 30 min of the OGTT for insulin (pmol/l) and glucose (mmol/l) as calculated using the trapezoidal method, respectively.

### Dietary pattern analysis

DPs were derived from the 40 food groups using the RRR method [15]. This method constructs uncorrelated linear combinations of food group intakes that maximize the explained variation in the intermediate disease-related variables. HIRI and MISI were selected as intermediate continuous variables. In RRR, the maximum number of extractable factors is constrained by the number of response variables. As two intermediate markers were included in the model (HIRI and MISI), a maximum of two DPs could be derived. Each factor was characterized by 40 factor loadings, corresponding to the 40 predefined food groups. Factor loadings represent the correlations of each food group with the DP score, where factor loadings with positive values indicate that the corresponding food groups are positively associated with the DP, and negative values indicate an inverse association. A larger factor loading value indicates that food group makes a greater contribution to the DP. Food groups with absolute loadings values superior to 0.2 were considered as contributing highly to the pattern [15]. DP scores, indicating the degree to which the participant’s diet conforms to the pattern, were computed at the individual level by summing the observed standardized food intakes per selected food group weighted according to the factor loadings.

### Statistical analysis

#### Data description

Results were expressed as mean ± standard deviation (SD) for quantitative variables (e.g. age, BMI, blood pressure, tissue specific IR indexes). Frequencies and percentages were used to summarize qualitative variables (e.g. sex, smoking status, glucose status). The conditions for linear model analysis were checked (normality and homoscedasticity of residuals).

Pearson’s correlation coefficients were used to assess the relationship between DP scores and nutrient intakes of the participants. Descriptive characteristics of the participants were reported as means and standard deviations or percentages across tertiles of the derived DP. The reported p-values refer to the one-way ANOVA test or chi-squared test, as appropriate.

#### Association between DP and insulin sensitivity/resistance measures

A logarithmic transformation was applied to all insulin sensitivity/resistance measures below to respect conditions for linear model analysis (normality and homoscedasticity of residuals). Linear regressions were performed to assess the relationship between RRR-derived DPs (in tertiles and as continuous variable) and subsequent insulin sensitivity/resistance measures: HOMA-IR, HOMA-β, Matsuda index, and disposition index. Four models were designed, with the first (M1) being univariate. Model 2 (M2) was adjusted for age, sex, and study center; model 3 (M3) was additionally adjusted for level of education, smoking and employment status; and model 4 (M4) was additionally adjusted for BMI. Covariates were selected a priori based on their established associations with dietary behaviors and metabolic risk. Study center was included due to the multicenter design and observed differences in glucose metabolism parameters between centers. BMI was added in the final model as a key determinant of insulin resistance and to further limit the potential impact of dietary misreporting, as described above. Because all food groups used to derive the dietary patterns were previously adjusted for total energy intake using the residual method, additional adjustment for total energy intake in the regression models was not performed, as the dietary pattern score already reflects energy-independent food composition. Results were presented as linear regression coefficients (β) with standard error (SE). Statistical analyses were performed using SAS statistical software 9.4 (© SAS Institute Inc., Cary, NC, USA). Results were considered significant at the 5% critical level (*p* < 0.05).

#### Sensitivity analysis

To investigate the robustness of the DP, we repeated RRR analysis excluding participants with BMI < 25 or ≥ 40 kg/m^2^ to be as close as possible to the selection criteria of the PERSON study. The resulting DPs were similar (data not shown).

## Results

Following dietary intake data analysis on 40 food groups, two DPs were extracted. The first DP explained 2.8% of the variation in all food groups, 13.7% of the variation in HIRI, 2.8% of the variation in MISI, and 8.2% of the total variation in the two selected tissue-specific insulin sensitivity indices. The second DP explained rather low cumulated variance (2.4%) in HIRI and MISI and was therefore not further considered in the subsequent analyses.

**Fig. 2:**
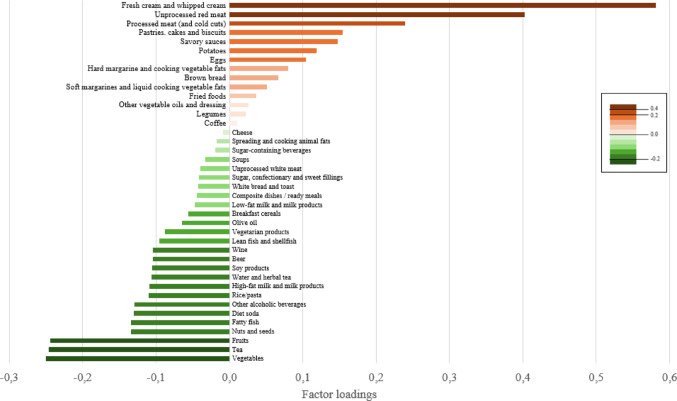
Reduced-rank regression factor loadings for the first dietary pattern (“Meat- and cream-based dietary pattern”) in the PERSON study (*n* = 700)

Percentage of variance explained and factor loadings of food groups on the DP are presented in Supplementary Table 2. The retained DP factor was characterized by high intakes of fresh cream and whipped cream, unprocessed red meat, and processed meat (and cold cuts) and low intakes of vegetables, tea, and fruits (Fig. 2). Accordingly, it was labelled a “Meat- and cream-based (low plant foods) dietary pattern”. The meat- and cream-based dietary pattern score was significantly associated with higher HIRI (univariate β-coefficient ± standard error: 0.13 ± 0.02, *p* < 0.001) and with lower MISI (univariate β-coefficient ± standard error: -0.14 ± 0.03, *p* < 0.001).

Adherence to the “Meat- and cream-based” dietary pattern was significantly and positively associated with intakes of animal proteins, and energy from total fats and all fatty acid classes. Moreover, it was negatively correlated to energy from carbohydrates, and intakes of simple sugars, plant proteins, dietary fibers, alcohol, and most of studied vitamins and minerals (Table 1).

**Table 1:** Pearson’s correlation coefficients between the DP and nutrient intakes expressed in grams per day or percent energy intake (*n* = 700)

		DP
Energy	Energy (kJ)	− 0.007
Nutrients	Total proteins (%)	0.002
	Plant proteins (g)	− 0.233**
	Animal proteins (g)	0.137*
	Total fats (%)	0.320**
	Saturated fatty acids (%)	0.326**
	Monounsaturated fatty acids (%)	0.159**
	Polyunsaturated fatty acids (%)	0.147**
	Trans fatty acids (%)	0.366**
	Total carbohydrates (%)	− 0.196**
	Mono- and di-saccharides (g)	− 0.181**
	Dietary fibers (g)	− 0.356**
	Alcohol (g)	− 0.096*
Minerals and vitamins	Sodium (mg)	− 0.045
	Calcium (mg)	− 0.190**
	Iron (mg)	− 0.182**
	β-carotene (µg)	− 0.327**
	Vitamin C (mg)	− 0.298**
	Vitamin E (mg)	− 0.011

* *p* < 0.05; **p < 0.001

The characteristics of the participants are shown according to the tertiles of the “Meat- and cream-based” dietary pattern score in Table 2. “Meat- and cream-based” dietary pattern was significantly associated with higher BMI and higher waist circumference, higher blood pressure, and higher fasting insulin levels. In addition, this DP was more adopted by subjects with less formal education or without nutritional supplementation. Habitual intakes related to each of the 40 food groups included in dietary patterns analysis are shown in Supplementary Materials (Supplementary Table 3).

**Table 2:** Sociodemographic characteristics, lifestyle characteristics and glucose metabolism of participants across the tertiles of the reduced rank regression-extracted “Meat- and cream-based” dietary pattern score (max *n* = 700)

Variable	Tertile 1	Tertile 2	Tertile 3	*p*-value
*Sociodemographic characteristics*				
Age (years)	61 ± 8	61 ± 8	61 ± 9	0.65
Women (%)	138 (59.7)	138 (59.7)	120 (50.4)	0.06
BMI (kg/m^2^)	29.2 ± 3.3	29.8 ± 3.5	31.1 ± 4.0	**< 0.0001**
Waist circumference (cm)	99.0 ± 10.9	100.2 ± 9.7	104.4 ± 10.7	**< 0.0001**
Employment status				0.35
Paid job	117 (50.7)	119 (51.5)	107 (45.0)	
Retired/pre-retired	81 (35.1)	80 (34.6)	84 (35.3)	
Other	33 (14.3)	32 (13.9)	47 (19.8)	
Level of education				**< 0.0001**
Low	21 (9.2)	31 (13.8)	60 (25.8)	
Intermediate	77 (33.8)	67 (29.8)	83 (35.6)	
High	130 (57.0)	127 (56.4)	90 (38.6)	
*Lifestyle characteristics*				
Smoking status				0.11
Non-smoker	114 (50.0)	112 (49.6)	143 (60.6)	
Former smoker	110 (48.3)	111 (49.1)	91 (38.6)	
Smoker	4 (1.8)	3 (1.3)	2 (0.9)	
Medication*, yes (%)	127 (55.0)	120 (52.0)	139 (58.4)	0.37
Nutritional supplement(s), yes (%)	122 (53.0)	104 (45.0)	97 (40.8)	**0.03**
Systolic blood pressure (mmHg)	130 ± 16	133 ± 17	134 ± 15	**0.03**
Diastolic blood pressure (mmHg)	80 ± 11	81 ± 11	83 ± 11	**0.004**
Total energy intake (kJ/day)	9459 ± 2797	9201 ± 2257	9552 ± 2602	0.31
*Glucose metabolism*				
Glucose status (%)				0.54
NGT	174 (75.3)	172 (74.5)	160 (67.2)	
IFG	12 (5.2)	11 (4.8)	16 (6.7)	
IGT	23 (10.0)	25 (10.8)	38 (16.0)	
Combined IFG/IGT	9 (3.9)	9 (3.9)	12 (5.0)	
T2DM	13 (5.6)	14 (6.1)	12 (5.0)	
Fasting glucose (mmol/L)	5.5 ± 0.7	5.6 ± 0.7	5.6 ± 0.7	0.27
Fasting insulin (pmol/L)	56.7 ± 42.6	58.7 ± 31.8	70.9 ± 41.0	**< 0.0001**
*Response variables*				
HIRI (AU)	438 ± 320	499 ± 335	603 ± 516	**< 0.0001**
MISI (AU)	0.152 ± 0.116	0.144 ± 0.115	0.121 ± 0.128	**< 0.0001**

Data are presented as mean ± SD, or number (%). NGT, normal glucose tolerance, IFG, impaired fasting glucose; IGT, impaired glucose tolerance T2DM, type 2 diabetes mellitus; HIRI, hepatic insulin resistance index; MISI, muscle insulin sensitivity index. *Medication corresponds to the use of medication that affects glucose and/or lipid metabolism, such as antidepressants, antihypertensives, anti-inflammatory medication, and statins. “Meat- and cream-based” dietary pattern scores were categorized into tertiles, with cut-off values defined as < –0.4262 for the first tertile, –0.4262–0.4369 for the second tertile, ≥ 0.4369 for the third tertile. Bold represents *p*-value < 0.05.

β-coefficients for insulin sensitivity/resistance measures according to the DP (in tertiles and as continuous variable), before and after multi-adjustment are shown in Table 3. In the unadjusted model, higher score on the “Meat- and cream-based” dietary pattern was associated with higher HOMA-IR and HOMA-β, and lower Matsuda index. These relationships remained statistically significant in the fully adjusted models. A comparison of R-squared and AIC values is provided in the Supplementary Materials (Supplementary Table 4). All fully adjusted models (M4) had the highest R-squared and AIC values.

**Table 3:** Associations between the “Meat- and cream-based” dietary pattern score (in tertiles and as continuous variable) and measures of insulin sensitivity and pancreatic β-cell function (*n* = 700)

Glucose metabolism	mean ± SD	M1		M2		M3		M4	
		β ± SE	*p*-value	β ± SE	*p*-value	β ± SE	*p*-value	β ± SE	*p*-value
HOMA-IR			**< 0.0001**		**0.0002**		**0.0013**		0.21
Tertile 1	2.1 ± 1.7	–		–		–		–	
Tertile 2	2.1 ± 1.3	0.08 ± 0.05		0.06 ± 0.05		0.06 ± 0.05		0.03 ± 0.05	
Tertile 3	2.6 ± 1.8	0.26 ± 0.05		0.20 ± 0.05		0.19 ± 0.05		0.09 ± 0.05	
Continuous variable		0.10 ± 0.02	**< 0.0001**	0.08 ± 0.02	**< 0.0001**	0.07 ± 0.02	**< 0.0001**	0.04 ± 0.02	**0.0059**
HOMA-β			**< 0.0001**		**0.0004**		**0.002**		0.13
Tertile 1	82.7 ± 61.2	–		–		–		–	
Tertile 2	85.0 ± 43.5	0.05 ± 0.05		0.04 ± 0.05		0.04 ± 0.05		0.02 ± 0.04	
Tertile 3	99.9 ± 55.3	0.20 ± 0.05		0.17 ± 0.05		0.16 ± 0.05		0.09 ± 0.04	
Continuous variable		0.08 ± 0.02	**< 0.0001**	0.07 ± 0.02	**< 0.0001**	0.07 ± 0.02	**< 0.0001**	0.05 ± 0.01	**0.0007**
Matsuda index			**< 0.0001**		**< 0.0001**		**< 0.0001**		**0.03**
Tertile 1	14.6 ± 8.8	–		–		–		–	
Tertile 2	13.1 ± 8.1	− 0.10 ± 0.06		− 0.08 ± 0.06		− 0.08 ± 0.06		− 0.05 ± 0.05	
Tertile 3	10.9 ± 7.3	− 0.31 ± 0.06		− 0.26 ± 0.06		− 0.25 ± 0.06		− 0.14 ± 0.05	
Continuous variable		− 0.13 ± 0.02	**< 0.0001**	− 0.11 ± 0.02	**< 0.0001**	− 0.11 ± 0.02	**< 0.0001**	− 0.08 ± 0.02	**< 0.0001**
Disposition index			0.12		0.40		0.39		0.70
Tertile 1	422 ± 210	–		–		–		–	
Tertile 2	435 ± 260	0.005 ± 0.05		0.02 ± 0.05		0.03 ± 0.05		0.04 ± 0.05	
Tertile 3	388 ± 221	− 0.090 ± 0.05		− 0.05 ± 0.05		− 0.05 ± 0.05		− 0.003 ± 0.05	
Continuous variable		− 0.02 ± 0.02	0.18	− 0.01 ± 0.02	0.69	− 0.01 ± 0.02	0.63	0.004 ± 0.02	0.81

Data are unadjusted β-coefficients ± standard error (M1) and multivariate-adjusted β-coefficients ± standard error (M2, M3 and M4). Model 2 (M2) was adjusted for age, sex, and study center; model 3 (M3) was additionally adjusted for level of education, smoking and employment status; and model 4 (M4) was additionally adjusted for BMI. “Meat- and cream-based” dietary pattern score was categorized into tertiles, with cut-off values defined as < −0.4262 for the first tertile, −0.4262–0.4369 for the second tertile, ≥ 0.4369 for the third tertile. Bold represents *p*-value < 0.05.

## Discussion/conclusion

In this study, we used the RRR method to identify at least one DP impacting the risk of type 2 diabetes, encompassing mechanisms related to tissue-specific IR. For this purpose, we have chosen to include both HIRI and MISI, two indices that reflect hepatic insulin resistance and muscle insulin sensitivity, respectively, in individuals without type 2 diabetes [22]. Only the first tissue-specific IR-related DP was retained as it accounted for the greatest variation in the selected intermediate markers.

Based on the proportions of variance explained, two key findings emerge from our results. Firstly, the fact that the DP explains a substantially greater proportion of the variance in HIRI (≈ 14%) compared to MISI (≈ 3%) suggests a differential association of this dietary pattern with tissue-specific IR. This finding indicates that the DP identified is more strongly related to hepatic IR than to skeletal muscle insulin sensitivity. While the underlying biological mechanisms remain to be elucidated, these differences might involve variations in lipid metabolism, inflammatory responses, or gut microbiota composition [39, 40]. However, given the cross-sectional nature of the study, these results should not be interpreted as evidence that diet preferentially influences hepatic rather than muscle insulin resistance. It is also plausible that underlying metabolic characteristics, including tissue-specific insulin resistance, may shape dietary behaviors or food choices. For example, individuals with impaired glucose metabolism may have already modified their diet, consciously or not, which could partly contribute to the observed associations.

Collectively, these findings support the relevance of considering tissue-specific pathways when investigating the relationship between diet and impaired glucose homeostasis. Integrating tissue-specific metabolic markers into personalized dietary recommendations may improve strategies for diabetes prevention and management.

Secondly, and interestingly, the total variance explained in HIRI and MISI (≈ 8%) was higher than what has been reported in previous RRR studies using metabolic or inflammatory biomarkers as response variables. However, several methodological differences between our study and earlier RRR applications should be considered before attributing this difference solely to the choice of biomarkers.

First, many previous studies used fasting glucose, HbA1c, HOMA-IR, circulating fatty acids, or inflammatory markers as response variables [11, 16, 29, 41–44]. These markers reflect global or downstream metabolic disturbances and do not distinguish between tissue-specific IR, in contrast to HIRI and MISI which specifically capture hepatic and skeletal muscle IR. Second, differences in population characteristics may have influenced the ability of RRR to extract discriminant dietary patterns. Most previous studies were conducted in general population samples with a wide BMI range and often included individuals with and without type 2 diabetes. In contrast, our study focused exclusively on adults with overweight or obesity without diagnosed type 2 diabetes, a metabolically at-risk but pre-diabetic population, potentially providing greater contrast in early IR phenotypes.

Third, differences in dietary data handling should be considered. In the majority of the earlier RRR studies [11, 16, 29, 41, 42], food groups were not energy-adjusted prior to DP derivation, whereas in our study all food groups were energy-adjusted using the residual method before RRR, allowing the extracted DP to reflect food composition independent of total energy intake.

Taken together, these methodological aspects may have enhanced the ability of RRR to identify a DP strongly linked to the selected response variables. Within this context, the use of HIRI and MISI—capturing tissue-specific IR rather than global metabolic markers—may further improve the sensitivity of RRR to detect diet–metabolism relationships compared with more commonly used biomarkers such as fasting glucose or HOMA-IR.

The DP extracted from the present dietary intake data was characterized by a higher consumption of fresh cream and whipped cream, unprocessed red meat, and processed meat (and cold cuts) but a lower consumption of vegetables, tea, and fruits. Despite the diversity of the biomarkers used in previous similar studies, commonalities in the identified dietary patterns emerge, such as high consumption of red meat and processed meat, along with low intake of fruits and vegetables, all of which have been linked to an increased risk of type 2 diabetes [16, 29, 42–45].

Some dietary components have also been shown to influence metabolic processes that predominantly take place in the liver, providing a relevant mechanistic context for interpreting our tissue-specific findings. Especially, both high-protein and high-fiber diets [46, 47], as well as the Mediterranean diet [46, 48], have been shown to reduce liver fat content, which in turn may improve hepatic insulin sensitivity [49, 50]. Although the dietary pattern identified here was not specifically characterized by macronutrient intake, it reflected lower consumption of foods rich in fibers (i.e. fruits and vegetables), features that are consistent with dietary factors known to affect hepatic metabolism.

Importantly, our identified dietary patterns also display distinct features. Notably, sugary beverages and refined grains did not play a significant role in shaping our dietary patterns, despite being frequently identified as key contributors in previous RRR-based studies, including a meta-analysis using various diabetes-related biomarkers as response variables [17]. This discrepancy with the existing literature prompted consideration of potential methodological explanations. One plausible explanation related to dietary misreporting, which is well documented for sugary foods and beverages, particularly in populations with overweight or obesity. Support for this interpretation comes from the NQplus study, which used a FFQ developed by the same research group and with a structure comparable to that used in the present study [51]. In NQplus, validation analyses incorporating objective urinary biomarkers of sugar intake (total urinary sucrose and fructose excretion) demonstrated underreporting of self-reported sugar intake [51]. Although we implemented several strategies to reduce the impact of misreporting, including exclusion of energy under- and over-reporters and energy adjustment of dietary variables, some degree of measurement error may persist.

Future studies incorporating objective biomarkers of sugar intake (e.g. total urinary excretion of sucrose and fructose [52]) may help clarify the contribution of sugary foods and beverages to dietary patterns related to insulin resistance.

Additionally, fresh and whipped cream emerged as distinctive components of our dietary pattern. Although the mean intake of these products was relatively low in the study population (approximately 3 g/day; see Supplementary Table 3), this finding remains noteworthy. In dietary pattern analyses, the contribution of a food group is not driven by absolute intake levels alone, but by its ability to discriminate between individuals and to covary with metabolic response variables. Even foods consumed in small amounts may therefore play a meaningful role if their intake is sufficiently heterogeneous or reflects specific dietary behaviours. One possible explanation is that fresh and whipped cream may be consumed frequently in small quantities throughout the day, for example when added to coffee, desserts, or snacks, resulting in a low average intake but a distinct habitual exposure. In this context, cream intake may also act as a marker of other dietary practices, such as consumption of sweet snacks or grazing behaviours, which could contribute to altered tissue-specific insulin resistance. Evidence from previous Dutch studies further helps contextualize our findings. In a prospective analysis, average cream intake was estimated at approximately 1 g/day and was not associated with prediabetes risk [53]; however, cream intake levels were lower than in our study population and may have provided limited contrast. In other studies examining dairy consumption and type 2 diabetes risk, cream was grouped together with desserts or other dairy products, preventing assessment of its specific metabolic effects [54].

More broadly, several studies have highlighted that cream is often excluded from, or inconsistently classified within, the “dairy products” category. For example, Wagner et al. noted that sources of dairy fat such as butter and cream are frequently omitted from dairy classifications in cardiometabolic research [55]. Similarly, an overview reported that, among 12 included reviews, only three assessed cream intake separately, and no association was observed [56]. These observations suggest that the potential role of cream products in metabolic health may have been largely overlooked rather than conclusively ruled out.

By analyzing fresh and whipped cream separately from other dairy products, our RRR-based approach allowed us to identify associations that may have been obscured in earlier studies [56]. Notably, milk and other dairy products were negatively correlated with the Meat- and cream-based dietary pattern score (although not major contributors to the DP), suggesting potentially distinct metabolic effects. Taken together, these findings support the hypothesis that fresh and whipped cream may exert different metabolic influences compared with other dairy products, particularly with respect to hepatic insulin resistance.

The identified DP was associated with worse glucose homeostasis and a higher prevalence of impaired glucose homeostasis (IFG and/or IGT), even after accounting for confounders such as sociodemographic factors and lifestyle characteristics. More specifically, higher DP scores were significantly associated with increased HOMA-IR and decreased Matsuda index. The present study contributes to the growing body of evidence highlighting dietary pattern as a key risk factor for type 2 diabetes, by exploring alternative pathophysiological pathways through which diet may influence disease risk. Notably, we employed the RRR approach to investigate the relationship between diet and tissue-specific IR phenotypes. Recent evidence suggests that populations can be divided into subgroups based on metabolic phenotyping, with each subgroup potentially benefiting from targeted dietary interventions that may be more effective than standard dietary recommendations. For example, large inter-individual variability has been observed in postprandial glycemic, insulinemic and lipemic responses to identical meals, driven by differences in blood measures, gut microbiota composition, and lifestyle factors. These studies have demonstrated that individuals can be stratified into metabolic response profiles, allowing prediction of personalized dietary responses [57, 58].

Insulin resistance can develop simultaneously in multiple tissues, but many individuals may exhibit more pronounced insulin resistance in either the liver or skeletal muscle. These distinct IR phenotypes (i.e. LIR and MIR phenotypes) have been linked to different cardiometabolic risk profiles, including distinct circulating lipidomic [59] and metabolomic profiles [60], adipose tissue transcriptome, and systemic inflammatory profile [60, 61]. Furthermore, we recently provided the proof-of-concept that these phenotypes may respond differently to dietary macronutrient manipulation (i.e. manipulation of the ratios of carbohydrates, proteins, fats, and fibers), influencing outcomes related to insulin sensitivity and cardiometabolic health [62]. Therefore, further investigation of the diet in relation to these metabolic phenotypes could provide valuable insights for developing more personalized dietary interventions.

Overall, our results suggest that dietary profiles characterized by higher intakes of unhealthy animal-based foods, such as red meat and processed meats, and a lower intake of health-promoting plant-based foods, including vegetables, fruits and tea, are associated with less favorable tissue-specific IR measures. While our analysis did not identify a strictly plant-based dietary pattern, the contrast underlying the identified DP reflects a dietary gradient opposing animal-based and plant-based food choices.

Interpreted in this light, individuals with lower DP scores would tend to consume relatively more fruits, vegetables, and tea and less meat and cream, which aligns with key features commonly observed in plant-rich DPs.

These findings align with a growing body of evidence emphasizing the positive impact of plant-rich diets on glycemic control and the reduction of type 2 diabetes risk [63, 64]. Diets high in plant-based foods—particularly those rich in fiber, antioxidants, and phytonutrients and poor in saturated fats—have been linked to weight loss, improved insulin sensitivity, reduced inflammation and improved gut microbial composition [65]. Collectively, these observations support the relevance of dietary shifts favoring greater plant food intake and reduced consumption of certain animal-based products in the context of insulin resistance prevention.

### Strengths and limitations

A strength of the present study is that our dietary patterns were not confounded by significant weight loss, since participants with weight change >3 kg in the last 3 months before the screening were excluded from the study. In addition, a rigorous method was used to assess the response variables. Estimates for hepatic and muscle insulin resistance from OGTT have been validated again the gold standard hyper insulinemic-euglycemic clamp technique. Finally, we adjusted for multiple confounders and conducted sensitivity analyses to confirm the robustness of the derived dietary patterns and associations with glucose metabolism disorders.

Our study, however, also has some limitations. One limitation is the cross-sectional study design, which prevented us from examining the association between the identified dietary patterns and incident cases of type 2 diabetes. However, we used well-established surrogate indices of insulin sensitivity and pancreatic β-cell function. While we could not assess causality due to the study design, these indices provide valuable insights into the potential metabolic effects of dietary patterns, offering an indirect yet robust measure of how these patterns might influence early risk stages of type 2 diabetes or cardiovascular diseases. Note that the identification of major food contributors to the extracted DP was based on crude analyses without adjustment for sociodemographic characteristics or lifestyle characteristics. While this approach was important to identify the DP, it does not account for potential confounding factors that may influence their individual contribution with HOMA-IR. Finally, the characteristics of the participants may affect the generalizability of our dietary patterns. The average age of the participants was 61 years, with a significant proportion having a high level of education and the majority being non-smokers. These factors could limit the representativeness of the identified dietary patterns, as they may not be fully applicable to younger or less educated populations, or those with different health behaviors, such as smoking.

### Implications and future research

Our findings open several perspectives for future research. First, they highlight the value of integrating tissue-specific IR markers, such as HIRI and MISI, into dietary pattern analyses to better understand the metabolic pathways linking diet to type 2 diabetes risk. Future longitudinal studies are needed to confirm whether DPs identified through these intermediate phenotypes predict incident diabetes more accurately than patterns derived from traditional biomarkers such as fasting glucose or HOMA-IR.

Second, the identification of distinct food groups, such as fresh and whipped cream, underscores the importance of examining specific foods separately rather than grouping them into broad categories, particularly in RRR approaches. This may help uncover associations that have been overlooked in previous research.

Third, our results support the relevance of metabolic phenotyping in nutrition research. Investigating how individuals with predominant hepatic or muscle IR respond differently to dietary exposures may contribute to the development of more targeted and personalized dietary recommendations.

### Conclusion

In this study, we used a RRR approach to derive dietary patterns linked to tissue-specific IR in adults with overweight or obesity. By incorporating HIRI and MISI as response variables, we explored an alternative pathway connecting diet to type 2 diabetes risk through hepatic and muscle insulin resistance.

The identified DP, characterized by higher intakes of fresh cream, whipped cream, unprocessed red meat, and processed meat, alongside lower intakes of fruits, vegetables and tea, was more strongly associated with hepatic than muscle insulin resistance. This finding highlights differential associations between diet and tissue-specific insulin resistance phenotypes and supports the relevance of considering tissue-specific metabolic pathways when investigating diet–diabetes relationships.

These results contribute to the growing field of metabolic phenotyping and suggest that integrating tissue-specific markers into dietary research may help refine more personalized strategies for diabetes prevention and management.

## Supplementary Information

Below is the link to the electronic supplementary material.

Supplementary Material 1

Supplementary Material 2

Supplementary Material 3

Supplementary Material 4

## Data Availability

Data described in the manuscript, code book, and analytic code will be made available upon request pending (Ellen E Blaak, *e.blaak@maastrichtuniversity.nl*).

## Abbreviations

BMR: Basal metabolic rate
DP: Dietary pattern
EI: Energy intake
FFQ: Food frequency questionnaire
HIRI: Hepatic insulin resistance index
NGT: Normal glucose tolerance
IFG: Impaired fasting glucose
IGT: Impaired glucose tolerance
IR: Insulin resistance
LIR: Liver insulin resistance
MIR: Muscle insulin resistance
MISI: Muscle insulin sensitivity index
RRR: Reduced rank regression
