# Supplementary material for: Differential associations of diet with hepatic and muscle insulin resistance: insights from an dietary pattern analysis in the PERSON study
Source: Eur J Nutr. 2026 May 26;65(4):142. doi: 10.1007/s00394-026-03996-8 (PMC13212402; doi:10.1007/s00394-026-03996-8)
Supplement: Supplementary file 1 — Supplementary Material 1 [file 394_2026_3996_MOESM1_ESM.docx]

**Supplementary Table 1** - Food groups included in dietary pattern analysis

| **Foods or Food groups to include in dietary pattern analysis** | **Included food items** |
| --- | --- |
| 1. White bread and toast | crispbread, or crackers; white rolls, currant buns, or muesli buns; white bread, raisin bread, currant bread, or muesli bread |
| 1. Brown bread | brown rolls, wholemeal rolls, multigrain rolls; brown bread, multigrain bread, wholemeal bread, rye bread; gingerbread |
| 1. Breakfast cereals | breakfast cereals (muesli, cornflakes, rice crispies, etc.) |
| 1. Rice/pasta | pasta or other pasta products, rice |
| 1. Potatoes | boiled potatoes or mashed potatoes (also in stews) |
| 1. Fried foods | French fries, fried or baked potatoes, potato slices, croquettes, rösti, etc.; fish sticks, fish fingers or kibbeling; fried savory snacks (croquettes, sausage rolls, bitterballen); chips or pretzels |
| 1. Fruits | citrus fruit, apples, bananas, other fresh fruit, kiwis, strawberries |
| 1. Vegetables | onion; cauliflower and broccoli, cabbage varieties (white, red, pointed, green, savoy, Chinese, farmer's and sauerkraut and Brussels sprouts), spinach, green beans, string beans and broad beans, carrots other cooked or stir-fried vegetables; cabbage varieties, lettuce, tomatoes, carrots, other raw vegetables |
| 1. Soups | soup with legumes, soup without legumes |
| 1. Legumes (excluding peas and broad beans; these are filled in with vegetables) | Legumes |
| 1. Unprocessed red meat | beef steak, beef tartare, beef chuck, beef roast, beef roast beef, beef entrecôte, beef sausage, beef chuck steak, beef rib steak, marbled beef steak, other beef; pork tenderloin, pork schnitzel, pork fricandeau, pork ham, pork chop (shoulder, rib, and tenderloin), pork sausage or slavink, bacon or bacon bits, other pork;  minced meat (all types), hamburger, smoked sausage or frankfurter, other types of meat and game, liver |
| 1. Unprocessed white meat | chicken or other poultry |
| 1. Processed meat (and cold cuts) | liver sausage, liver spread, pâté, liver pie, liver cheese, Berliner, sausage types such as luncheon meat, roast minced meat, cervelat sausage, etc., other types of meat products, unknown types of meat products, cooked liver, smoked meat, fricandeau, roast beef, smoked pork loin, chicken breast, chicken roll, ham;  liver sausage, liver spread, pâté, liver pie, liver cheese, Berliner, sausage types such as luncheon meat, roast minced meat, cervelat sausage, etc., other types of meat products, smoked meat, fricandeau, roast beef, smoked pork chop, chicken breast, chicken roll, ham; salad without fish; salad without fish; non-fried savory snacks (croquettes, sausage rolls, bitterballen) |
| 1. Lean fish and shellfish | Lean fish such as cod, plaice, tilapia, pangasius, trout, tuna, etc., unknown types of fish;  shrimp, mussels, other shellfish; salad with fish; salad with fish |
| 1. Fatty fish | Fatty fish such as salmon, mackerel, eel, smoked or steamed fish (e.g., salmon, mackerel, herring) or sardines, canned or jarred herring, salted herring |
| 1. Eggs | eggs |
| 1. Soy products | soy milk, soy drink or soy dessert |
| 1. Vegetarian products | vegetarian meat products; quorn, valess, tofu/tempeh/soy products, other meat substitutes |
| 1. Composite dishes / Ready meals | pizza; oriental meal |
| 1. Low-fat milk and milk products | skim milk, semi-skimmed milk; low-fat (fruit) yogurt or (fruit) quark, semi-skimmed (fruit) yogurt or (fruit) quark; semi-skimmed milk (for coffee) |
| 1. High-fat milk and milk products | whole milk, buttermilk, unknown type of milk; whole (fruit) yogurt or (fruit) quark, unknown type of yogurt or quark; coffee milk or creamer; custard or pudding; ice cream or milk-based ice cream |
| 1. Fresh cream and whipped cream | whipped cream; fresh cream or other cooking cream |
| 1. Cheese | cheese spread or dairy spread, cream cheese or foreign cheese, unknown type of spreadable cheese;  20+ or 30+ cheese, 40+ or 48+ full-fat cheese, unknown type of sliceable cheese; 40+ or 48+ full-fat cheese (for hot meals); 40+ or 48+ full-fat cheese (for snacks) |
| 1. Spreading and cooking animal fats | Spreadable fats  butter, semi-skimmed butter;  Preparation/cooking fats  butter, lard or beef fat; Frying fat (solid) |
| 1. Hard margarine and cooking vegetable fats | Spreadable fats  margarine in tub, margarine in packet  Preparation/cooking fats  margarine in tub, margarine in packet, cooking and frying product from a packet, unknown type of cooking fat; Liquid frying product |
| 1. Soft margarines and liquid cooking vegetable fats | Spreadable fats  low-fat margarine or light margarine, diet low-fat margarine or diet light margarine, low-fat margarine with plant sterols/stanols, low-fat margarine product (25% fat or less), diet margarine;  Preparation/cooking fats  Diet margarine, cooking and frying product from a bottle, liquid margarine |
| 1. Olive oil | olive oil |
| 1. Other vegetable oils and dressing | sunflower oil, soybean oil, salad oil, etc.; dressing |
| 1. Savory sauces | gravy; satay sauce, tomato sauce, other hot sauces;  tomato ketchup or other red sauces,  mayonnaise, halvanaise, French fry sauce, or other non-red sauces, unknown type of sauce; tomato ketchup or other red sauces; mayonnaise, halvanaise, French fry sauce, or other non-red sauces, unknown type of sauce |
| 1. Nuts and seeds | seeds or kernels, nuts, nut mix, trail mix;  peanuts or cocktail nuts, nuts nut mix, trail mix |
| 1. Pastries, cakes, and biscuits | croissants; small cookies or biscuits; large cookies or slices of cake; nutritional biscuits (Liga, Sultana, etc.) or muesli bars; pastries or cakes; candy bars or chocolate candies; pancakes |
| 1. Sugar, confectionery, sweet fillings | peanut butter or nut butter; chocolate sprinkles or flakes, chocolate spread, hazelnut spread, chocolate butter, other sweet spreads; sugar (in coffee); sugar (for tea); bonbons;  milk chocolate, dark chocolate, white chocolate;  candy |
| 1. Water and herbal tea | water; herbal tea |
| 1. Sugar-containing beverages | orange juice, apple juice, other fruit juices;  fruit drinks, double drinks, or multi-fruit drinks;  tomato juice, tomato vegetable juice; soft drinks, fruit lemonade, sports drinks or energy drinks;  breakfast drinks; drinkable yogurt or other dairy drinks, chocolate milk, unknown type of dairy drink |
| 1. Diet soda | light soft drinks, light fruit lemonade |
| 1. Coffee | Coffee |
| 1. Tea | black tea, green tea |
| 1. Beer | beer; low-alcohol or non-alcoholic beer |
| 1. Wine | red wine, white wine, or rosé |
| 1. Other alcoholic beverages | sherry, port, vermouth, etc., spirits |
