# Supplementary material for: Differential associations of diet with hepatic and muscle insulin resistance: insights from an dietary pattern analysis in the PERSON study
Source: Eur J Nutr. 2026 May 26;65(4):142. doi: 10.1007/s00394-026-03996-8 (PMC13212402; doi:10.1007/s00394-026-03996-8)
Supplement: Supplementary file 2 — Supplementary Material 2 [file 394_2026_3996_MOESM2_ESM.pdf]

Supplementary Table 2 – Reduced-rank regression factor loadings for the first dietary pattern ("Animal-based" dietary pattern) and explained variation in the PERSON study (n=700).

	**DP**
White bread and toast	-0.0428
Brown bread	0.0662
Breakfast cereals	-0.0556
Rice/pasta	-0.1105
Potatoes	0.1186
Fried foods	0.0371
Fruits	-0.2442**
Vegetables	-0.2497**
Soups	-0.0332
Legumes	0.0222
Unprocessed red meat	0.4030**
Unprocessed white meat	-0.0392
Processed meat (and cold cuts)	0.2397**
Lean fish and shellfish	-0.0961
Fatty fish	-0.1340
Eggs	0.1045
Soy products	-0.1053
Vegetarian products	-0.0882
Composite dishes / ready meals	-0.0442
Low-fat milk and milk products	-0.0469
High-fat milk and milk products	-0.1089
Fresh cream and whipped cream	0.5810**
Cheese	-0.0089
Spreading and cooking animal fats	-0.0179
Hard margarine and cooking vegetable fats	0.0804
Soft margarines and liquid cooking vegetable fats	0.0513
Olive oil	-0.0643
Other vegetable oils and dressing	0.0256
Savory sauces	0.1476
Nuts and seeds	-0.1341
Pastries. cakes and biscuits	0.1542
Sugar, confectionary and sweet fillings	-0.0419
Water and herbal tea	-0.1062
Sugar-containing beverages	-0.0198

Diet soda	-0.1303
Coffee	0.0106
Tea	-0.2463**
Beer	-0.1044
Wine	-0.1041
Other alcoholic beverages	-0.1290
**Explained variation in food groups, %**	**2.7998**
**Explained variation in response variables, %**	**8.2441**
**Explained variation in HIRI, %**	**13.7388**
**Explained variation in MISI, %**	**2.7494**

**Factor loading >0.20 in absolute value.
