# Supplementary material for: Differential associations of diet with hepatic and muscle insulin resistance: insights from an dietary pattern analysis in the PERSON study
Source: Eur J Nutr. 2026 May 26;65(4):142. doi: 10.1007/s00394-026-03996-8 (PMC13212402; doi:10.1007/s00394-026-03996-8)
Supplement: Supplementary file 3 — Supplementary Material 3 [file 394_2026_3996_MOESM3_ESM.pdf]

Supplementary Table 3 - Food intakes (expressed in grams per day) related to each of the 40 food groups included in dietary patterns analysis (n=700).

Food groups	Mean ± SD
White bread and toast	17.5 ± 21.4
Brown bread	92.3 ± 61.9
Breakfast cereals	11.4 ± 18.4
Rice/pasta	58.0 ± 51.5
Potatoes	39.6 ± 36.3
Fried foods	39.5 ± 32.8
Fruits	170.2 ± 115.8
Vegetables	161.7 ± 111.7
Soups	53.6 ± 64.7
Legumes	25.2 ± 29.5
Unprocessed red meat	59.0 ± 37.0
Unprocessed white meat	12.3 ± 16.0
Processed meat (and cold cuts)	38.2 ± 44.0
Lean fish and shellfish	14.9 ± 16.1
Fatty fish	10.7 ± 11.0
Eggs	23.3 ± 19.7
Soy products	11.9 ± 52.6
Vegetarian products	5.9 ± 12.3
Composite dishes / ready meals	24.7 ± 32.1
Low-fat milk and milk products	157.8 ± 173.4
High-fat milk and milk products	116.6 ± 134.9
Fresh cream and whipped cream	3.2 ± 5.3
Cheese	37.5 ± 30.7
Spreading and cooking animal fats	5.6 ± 9.7
Hard margarine and cooking vegetable fats	3.4 ± 7.0
Soft margarines and liquid cooking vegetable fats	10.0 ± 12.2
Olive oil	2.2 ± 2.9
Other vegetable oils and dressing	5.1 ± 7.1
Savory sauces	26.1 ± 24.6
Nuts and seeds	18.0 ± 20.4
Pastries, cakes and biscuits	47.5 ± 34.4
Sugar, confectionary, and sweet fillings	29.6 ± 26.7
Water and herbal tea	738.8 ± 478.9
Sugar-containing beverages	140.9 ± 221.1
Diet soda	53.6 ± 140.9
Coffee	458.0 ± 263.6
Tea	228.4 ± 292.7
Beer	80.1 ± 133.7
Wine	49.6 ± 66.4

Other alcoholic beverages	6.2 ± 15.8
