# Supplementary material for: Differential associations of diet with hepatic and muscle insulin resistance: insights from an dietary pattern analysis in the PERSON study
Source: Eur J Nutr. 2026 May 26;65(4):142. doi: 10.1007/s00394-026-03996-8 (PMC13212402; doi:10.1007/s00394-026-03996-8)
Supplement: Supplementary file 4 — Supplementary Material 4 [file 394_2026_3996_MOESM4_ESM.pdf]

Supplementary Table 4 – Model fit statistics (R^2 and AIC) for the associations between the "Animal-based" dietary pattern score and measures of insulin sensitivity and pancreatic β-cell function (see Table 3).

	M1		M2		M3		M4	
**Glucose metabolism**	**R²**	**AIC**	**R²**	**AIC**	**R²**	**AIC**	**R²**	**AIC**
HOMA-IR								
Tertiles	0.03	1212.3	0.11	1174.0	0.10	1171.3	0.27	1041.3
Continuous variable	0.04	-788.3	0.12	-837.8	0.12	-824.1	0.28	-962.7
HOMA-β								
Tertiles	0.03	990.0	0.06	979.8	0.06	981.0	0.17	908.7
Continuous variable	0.05	-1018	0.08	-1039	0.09	-1021	0.19	-1100
Matsuda index								
Tertiles	0.04	1336.1	0.12	1294.3	0.11	1285.4	0.25	1175.8
Continuous variable	0.06	-672.2	0.13	-723.9	0.14	-715.8	0.27	-833.2
Disposition index								
Tertiles	0.003	1186.7	0.07	1152.7	0.07	1145.3	0.10	1131.8
Continuous variable	0.003	-806.8	0.08	-855	0.08	-844.4	0.11	-865.7

Four linear models were designed, with the first (M1) being univariate. Model 2 (M2) was adjusted for age, sex, and study centre; model 3 (M3) was additionally adjusted for level of education, smoking and employment status; and model 4 (M4) was additionally adjusted for BMI.
